# Peptide Conjugate on Multilayer Graphene Oxide Film for the Osteogenic Differentiation of Human Wharton’s Jelly-Derived Mesenchymal Stem Cells

**DOI:** 10.3390/polym13193290

**Published:** 2021-09-26

**Authors:** Perng Yang Puah, Pak Yan Moh, Coswald Stephen Sipaut, Ping Chin Lee, Siew Eng How

**Affiliations:** 1 Programme of Biotechnology, Faculty of Science and Natural Resources, Universiti Malaysia Sabah, Jalan UMS, Kota Kinabalu 88400, Sabah, Malaysia; pypuah@hotmail.com (P.Y.P.); leepc@ums.edu.my (P.C.L.); 2 Programme of Industrial Chemistry, Faculty of Science and Natural Resources, Universiti Malaysia Sabah, Jalan UMS, Kota Kinabalu 88400, Sabah, Malaysia; 3 Faculty of Medicine and Health Sciences, Universiti Malaysia Sabah, Jalan UMS, Kota Kinabalu 88400, Sabah, Malaysia; 4 Programme of Chemical Engineering, Faculty of Engineering, Universiti Malaysia Sabah, Jalan UMS, Kota Kinabalu 88400, Sabah, Malaysia; css@ums.edu.my; 5 Biotechnology Research Institute, Universiti Malaysia Sabah, Jalan UMS, Kota Kinabalu 88400, Sabah, Malaysia

**Keywords:** multilayer graphene oxide, peptide, Wharton’s jelly mesenchymal stem cells, osteogenic differentiation

## Abstract

Graphene oxide (GO) is extensively studied as a template material for mesenchymal stem cell application due to its two-dimensional nature and unique functionalization chemistries. Herein, a new type of peptide-conjugated multilayer graphene oxide (peptide/m-GO film) was fabricated and used as biomaterial for culturing human Wharton’s jelly-derived mesenchymal stem cells (WJ-MSCs). The characterization of the peptide/m-GO films was performed, and the biocompatibility of the WJ-MSCs on the peptide/m-GO films was investigated. The results demonstrated that the peptide conjugate on the m-GO film did not hamper the normal growth of WJ-MSCs but supported the growth of WJ-MSCs after the 6-day culture period. In addition, the osteogenic differentiation of WJ-MSCs on the peptide/m-GO films was enhanced as compared with the parent m-GO film. Therefore, such peptide-conjugated m-GO films could provide a highly biocompatible and multifunctional 2D material to tailor the potential application of WJ-MSCs in bone tissue regeneration.

## 1. Introduction

Mesenchymal stem cells (MSCs) have been studied extensively in regenerative medicine. MSCs can be either applied alone or integrated with scaffolds in the treatment of several diseases, including bone and cartilage dehiscence, wound therapy, and cardiovascular and neural disorders [1,2,3,4]. MSCs can be isolated from various sources, including bone marrow, adipose tissues, mobilized peripheral blood, skin epithelium, and umbilical cord [5]. The use of human Wharton’s jelly-derived mesenchymal stem cells (WJ-MSCs) isolated from human umbilical cord tissue, which is considered a medical waste discarded at birth, is noninvasive and involves no ethical concerns. Moreover, WJ-MSCs do not turn into teratogenic or carcinogenic cells upon transplantation, and they represent the intermediate state between adult and embryonic stem cells [6,7,8]. WJ-MSCs have been the subject of intensive research as a potential cell source for mesenchymal stem-cell-based therapy in the fields of tissue engineering and regenerative medicine [9,10]. However, in previous studies, WJ-MSCs were reported to have a lower osteogenic differentiation potential as compared with bone marrow-isolated mesenchymal stem cells (BM-MSCs) [11,12]. Thus, it is important to search for a topography that can instruct and maintain the differentiation of WJ-MSCs by cell-material interactions [13]. Recently, the environmental component of stem-cell-based therapy, especially graphene-based nanomaterials, has received significant attention for its role as a temporary extracellular matrix (ECM) for stem cell culture. Previous studies have suggested that graphene-based nanomaterials may be able to unlock the potential of stem-cell-based therapies [14,15]. Particularly, graphene oxide (GO) has been extensively studied for its adhesive ability and biocompatibility with MSCs to regulate the cell signaling that triggers cell responses, including cell proliferation and differentiation [14,16,17,18]. For example, the multilayer GO film prepared by Rosa et al. using a simple drop-casting method showed a good attachment and proliferation of dental pulp stem cells [19]. GO is a two-dimensional sheet of carbons, with edges bound to the carboxyl, epoxide, and hydroxyl groups [20]. With these properties, the GO surface possesses greater interactions with biomacromolecules via covalent or noncovalent interactions, such as *π*–*π* interactions, van der Waals forces, ionic interactions, and hydrogen bonding [21,22,23].

Considering the presence of reactive functional groups at the basal place and edges of GO, researchers have discovered that GO has a great ability to adsorb insulin and dexamethasone through noncovalent interactions [17,24,25], which provides an effective preconcentration platform for the growth and differentiation of human stem cells [14]. This has led to further exploration of the modification of the GO surface as a biomaterial for stem cells. Recent research has focused on designing or discovering any bioactive molecules that can functionalize the GO surface to stimulate or enhance the proliferation and differentiation of stem cells. For example, GO has been incorporated with lysozyme (Lys) and tannic acid (TA) [26], chitosan [27], polyethylene glycol (PEG)-based hydrogels [28], poly(lactic-*co*-glycolic acid) (PLGA) nanofiber [29], hydroxyapatite (HA)-sodium alginate (SA) [30], protein (silk fibroin) [31], and polypeptide (poly-L-lysine) [32] to improve the stem cell attachment, proliferation, and osteogenic differentiation. 

Synthetic biomaterials, such as peptides and polymers, are easy to fabricate and represent a reliable alternative to in vitro stem cell culture [33]. The utilization of synthetic peptides as a component within engineered biomaterials is now a standard technique [34,35,36,37]. Recently, researchers have focused on identifying and exploring the use of peptide and peptide derivatives as potential candidates for the development of 2D or 3D cell-culture materials to tailor the stem cell’s microenvironment to further improve the adhesion, proliferation, and differentiation of stem cells [38,39,40,41]. For instance, peptides have been grafted to a variety of synthetic polymers to endow the material with cell-adhesive, enzymatically degradable, and growth-factor-binding properties [42,43]. Previous studies have reported that adding short peptide bioactive sequences could enhance either the cell adhesion, cell proliferation, cell viability, or cell differentiation lineage. For example, previous studies have derived peptide sequences from collagen (DGEA) [44], laminin (IKVAV, YIGSR) [45], fibronectin (RGDSP, WQPPRARI) [46,47,48], enamel matrix derivative (WYQNMIR) [49], angiopoietin-1 (QHREDGS) [50], and heparin-binding domain (KRSR) [51]. Zhang and coworkers studied the binding strength of 20 amino acids with GO and found that positively charged site-chain amino acids bound with the GO surface through electrostatic interaction while binding with the amino acids His, Trp, Tyr, and Phe through π–π interaction due to the presence of the amino acids’ aromatic rings [52]. GO with carboxyl groups on its edges with pK_a_ = 5.2 appeared to be negatively charged at a physiological pH of about 7. Thus, the designed peptide should be positively charged at pH 7 for the efficient noncovalent binding of the peptide on the GO film [53].

In our previous study, we tested the biocompatibility of multilayer GO (m-GO) film toward WJ-MSCs [54]. The present work aimed to conjugate a peptide on the m-GO film through a self-assembly technique to improve the biocompatibility and osteogenic differentiation of the m-GO film toward WJ-MSCs. Hence, four peptide sequences were designed and conjugated with an m-GO film (peptide/m-GO film). The peptide/m-GO films were then characterized. Subsequently, the biocompatibility and osteogenic differentiation potential were investigated by culturing with WJ-MSCs. Specifically, we sought to determine whether the peptide conjugated with the m-GO film could act as a potential biocompatible substrate for WJ-MSCs with tailored properties.

## 2. Materials and Methods

### 2.1. Designation of Peptide Sequences

Peptide sequences were designed by combining the bioactive short peptide sequences. The short peptide sequences were combined according to the properties of each amino acid, as previously reported by Zhang and coworkers [52]. Briefly, peptide was noncovalently attached to the GO surface through the presence of a positively charged amino acid (via electrostatic force) and the aromatic rings of amino acids (via *π–π* interactions). Table 1 presents the characteristic of the peptide sequences that contributed to the interaction of the peptide with the GO film. Four types of peptide sequences formed by three different bioactive short peptide sequences, namely, PepS1, PepS2, PepS3, and PepS4, were purchased from Beijing SBS Genetech Co., Ltd. (Beijing, China) at >95% purity (HPLC purified). 

### 2.2. Fabrication of m-GO and Peptide/m-GO Film 

A multilayer graphene oxide (m-GO) film with 25 µg of GO loading prepared according to our previously reported method [54] was used as a substrate for the fabrication of GO/peptide films. Briefly, 100 µL of the GO flake solution (0.25 mg/mL) was added over the treated 10 mm glass coverslip and allowed to dry at 30 °C for 24 h. GO/peptide films were fabricated following the modified previously reported method [52]. Briefly, each peptide sequence (PepS1, PepS2, PepS3, or PepS4) was dissolved in distilled water at a concentration of 50 µM. Then, 100 µL of the peptide solution was dropped onto the m-GO film surface and incubated for 30 min. At the end of the incubation period, the peptide/m-GO films were rinsed twice with distilled water to remove the unabsorbed peptide and allowed to dry at 35 °C for 12 h.

### 2.3. Characterization of m-GO and Peptide/m-GO Film 

The 3D topography and thickness of both films were analyzed using a Dimension Edge atomic force microscopy (Bruker Corporation, Billerica, MA, USA) coupled with ScanAsyst-Air cantilevers (Bruker Corporation, Billerica, MA, USA) at a 0.4 N/m nominal spring constant. The measurement of the film’s surface roughness was calculated using NanoScope Analysis 1.7 software (Bruker Corporation, Billerica, MA, USA). The functional groups of the fabricated films were examined using Fourier-transform infrared (FTIR) spectroscopy (Model: Spectrum 100, Perkin Elmer, Waltham, MA, USA). The surface morphology and composition of both films were measured using an energy dispersive X-ray (EDX) spectroscopy on a scanning electron microscope (SEM) (Model: S-3400N, Hitachi, Tokio, Japan). 

### 2.4. WJ-MSC Culture

This study was approved by the Ethics and Research Committee of Universiti Malaysia Sabah with approval code JKEtika 1/16 (1). The human Wharton’s jelly mesenchymal stem cells (WJ-MSCs) from the umbilical cord matrix were kindly provided by Dr. Siti Fatimah Simat. The given WJ-MSCs were maintained as described by our previous report [55]. The cells were cultured in T25 flasks with a basal medium (BM), which consisted of Dulbecco’s modified Eagle’s medium, Nutrient Mixture F-12 (DMEM/ F-12; Gibco, Paisley, UK) supplemented with 10% FBS (Gibco, Paisley, UK), 1% antibiotic-antimycotic (Gibco, Paisley, UK), 1% glutamine (Gibco, Paisley, UK), and 1% L-ascorbic acid (Sigma-Aldrich, Saint Louis, MO, USA). Cells were maintained at 37 °C in a humidified 5% CO_2_ incubator and replaced with a fresh medium every 2 to 3 days until reaching 80%–90% confluency. When the cells reached confluency, they were detached from the plate by adding 0.125% trypsin–EDTA solution (Gibco, Paisley, UK). The cells were then incubated for 3 min at 37 °C incubator.

All the cell assays on the parent m-GO film and peptide/m-GO films were carried out using WJ-MSCs between passage 3 and passage 5. The films (*n* = 3) were sterilized with UV light for 20 min prior to the cellular studies [56]. The coated 10 mm films were placed into a 48-well plate with 10,000 WJ-MSC cells seeded. Prior to cell seeding on samples, a cell count was performed using a hemacytomer, and 350 µL BM was added to each well for cell proliferation. The cell samples were incubated at 37 °C in a humidified 5% CO_2_ incubator. The medium was changed every 3 days. 

### 2.5. Cell Viability Assay

An MTT assay was used to determine the cell viability of the WJ-MSCs’ growth on the parent m-GO film and peptide/m-GO films. After each incubation period (days 1, 3, and 6), 200 µL of the MTT solution was added to each well and left for 2 h for a cell response to take place. This was followed by the addition of 200 µL of dimethyl sulfoxide to dissolve the purple crystals reduced by the living cells. The optical density of 100 µL of the resulting solution was transferred into new 96-well plate and measured at 570 nm using an Infinite M200Pro microplate reader (Tecan, Grödig, Austria). 

### 2.6. Cell Morphology

The morphology of the WJ-MSCs on the parent m-GO film and peptide/m-GO films was observed using an inverted microscope Olympus IX73 (Olympus Corp., Tokyo, Japan). Prior to viewing by a microscope, the cell medium was discarded, and cells were washed with 1× phosphate-buffered saline (200 µL, PBS). Images of the WJ-MSCs incubated on films under the desired incubation period were captured with either 4×, 10×, or 40× magnification.

### 2.7. Osteogenic Differentiation

The parent m-GO film and peptide/m-GO films were placed in a 48-well plate, and WJ-MSCs were seeded at a density of 10,000 cells/well (*n* = 3) in BM. After 3 days of incubation, the cells were attached. Then, the medium was replaced with the BM or osteogenic medium (OM, StemPro osteogenesis differentiation kit;cat. no. A1007201; Life Technology, Carlsbad, CA, USA) according to the manufacturer’s directions. The induction medium was changed every 2–3 days, and the cultures were maintained for the next 14 days. On day 14, the cells were then washed with 1× PBS once and fixed in 300 µL of 10% formalin in PBS for 20 min at room temperature. After fixation, the well plate was washed three times with 1 × PBS and once with distilled water. Osteogenic-induced cells were stained with an Alizarin Red Solution Kit (Life Technology, Carlsbad, CA, USA). Prior to observation under an inverted microscope Olympus IX73 (Olympus Corp., Tokyo, Japan), the samples were washed with distilled water three times. The Alizarin Red-stained WJ-MSCs showed maximum expression as the calcium had reached maximum deposition.

### 2.8. Statistical Analysis

The numeric data for the MTT assay (*n* = 3) were analyzed using a one-way analysis of variance (ANOVA), followed by a *t*-test. Results were considered significant when *p* < 0.05. All the values were presented as ± standard error of the mean (SEM). 

## 3. Results and Discussion

### 3.1. Characterization of Multilayer GO and Peptide/m-GO Films

**Scheme 1:**
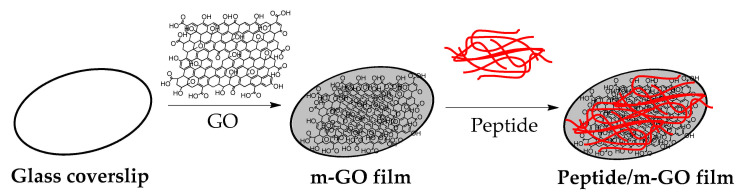
Schematic illustration of the preparation of peptide conjugated on m-GO film.

**Figure 1:**
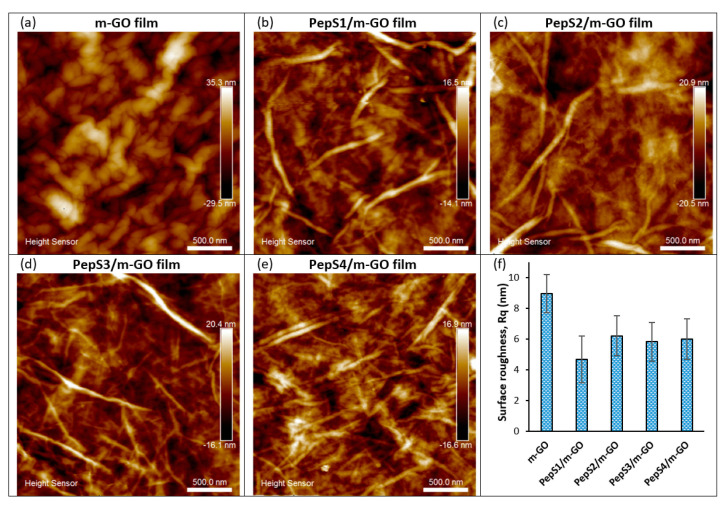
AFM topographic image of m-GO film (**a**) before and (**b**–**e**) after conjugation with peptide at 2.5 µm × 2.5 µm and corresponding surface roughness measurements (**f**).

The preparation process of the peptide conjugate on the multilayer GO (m-GO) films is shown in Scheme 1. The m-GO films were naturally formed by drying the aqueous solution of GO as previously reported [19,54]. Then, the designated peptide sequences (PepS1, PepS2, PepS3, and PepS4) with positively charged and aromatic rings interacted with the negatively charged m-GO film surface [52,57,58]. Figure 1 shows the AFM images of the m-GO and peptide-conjugated m-GO films. From Figure 1a, the wrinkled surface of the m-GO film can be observed. The wrinkles mainly resulted from the amphiphilicity of the GO flakes, which self-assemble and stack over one another to form the multilayer GO film as water is slowly evaporated [59,60]. Recent molecular dynamics simulation studies have shown that wrinkled surfaces on graphene-based materials possess greater interactions between peptides/proteins and graphenic basal planes owing to their greater surface area at the interface [61,62,63]. When the designated peptide sequences were noncovalently adsorbed onto the m-GO films, their surface morphology exhibited different trends (Figure 1b–e and Figure S1) and showed a decrease in surface roughness (mean square roughness, Rq) (Figure 1f). The AFM images obtained here can be disturbed by the specific interactions of the peptide with the negatively charged m-GO film’s surface or drying effects. Thus, the AFM images should not be overinterpreted [64,65,66]. However, the observed changes may be attributed to the fact that the m-GO film was formed through a facile self-assembly of GO nanosheets at the liquid/air interface by stacking the sheet through interlayered van der Waals forces [67,68]. Hence, the dissimilarity found in the AFM images clearly demonstrates the alterations of the physicochemical properties of the m-GO film before and after the conjugation with peptides. 

**Figure 2:**
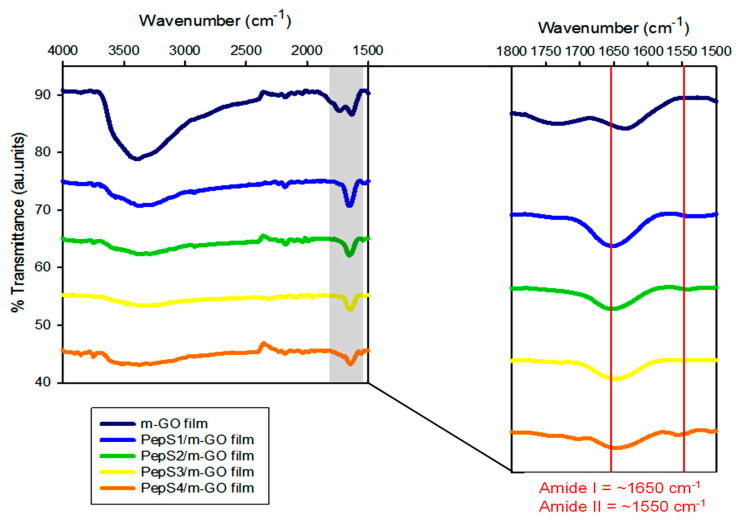
FTIR spectra of m-GO film and functionalized m-GO film with PepS1, PepS2, PepS3, and PepS4.

FTIR spectroscopy of the m-GO film and m-GO film conjugated with four different peptide sequences (PepS1, PepS2, PepS3, and PepS4) is shown in Figure 2 and Figure S2. In the FTIR spectroscopy of the m-GO film, characteristic peaks of C=O stretching confirmed the oxidized carboxyl group on the graphene surface and the presence of C=C alkene stretching. The sp2 carbon structure of the GO can be observed at 1729 and 1632 cm^−1^, respectively. A broad peak at 3395 cm^−1^ can be ascribed to the O–H vibrations of the hydroxyl group of the GO [54]. The FTIR spectra of the m-GO film conjugated with four different peptide sequences (PepS1, PepS2, PepS3, and PepS4) appear to be very similar, exhibiting one broad peak with weaker intensities at ~3390 cm^−1^ as compared with the m-GO film, which indicates that the O–H vibrations of the hydroxyl group on the surface of the m-GO film were diminished in the presence of the peptide. A strong amide I peak (C=O stretching vibrations of the peptide bonds) and a weaker amide II peak (C–N stretching vibrations in combination with N–H bending) were observed at 1650 and 1550 cm^−1^, respectively. The existence of amide peaks supports the presence of the peptide in all the peptide-conjugated m-GO films [69,70]. These amide peaks were not found in the parent material, the m-GO film. 

The presence of carbon (C), oxygen (O), and nitrogen (N) was identified in the parent m-GO film and all peptide-conjugated m-GO films (PepS1/m-GO, PepS2/m-GO, PepS3/m-GO, and PepS4/m-GO film) using energy dispersive X-ray (EDX) spectroscopy (Table 2). Different levels of carbon and oxygen were observed in different conjugations of the peptide onto the m-GO film. However, the presence of nitrogen was only observed in the peptide-conjugated m-GO film, which can be attributed to the side chains of the various amino acids of the peptide [70], hence leading to the adsorption of the peptide onto the m-GO film surface. Herein, considering that the characterization results of AFM, FTIR, and EDX were consistent with one another, these data explain the successful modification of the m-GO film with our designated peptide sequences (PepS1, PepS2, PepS3, and PepS4). 

### 3.2. WJ-MSC Morphology and Viability on the Multilayer GO and Peptide/m-GO Films

**Figure 3:**
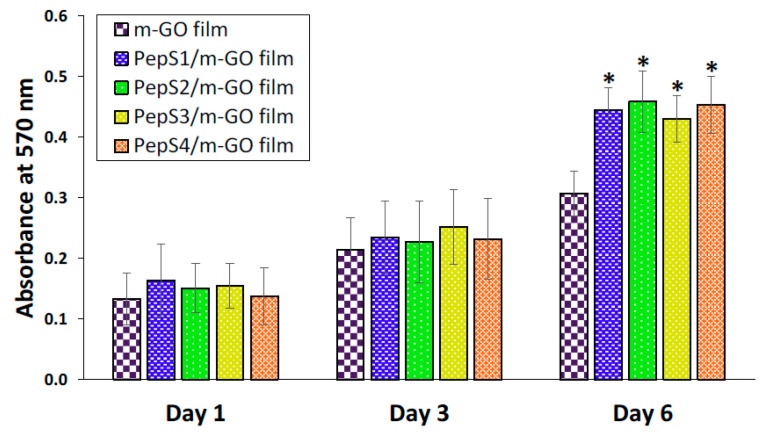
Cell viability of the WJ-MSCs evaluated at the m-GO film before and after conjugate with peptide sequences (PepS1, PepS2, PepS3, and PepS4) was quantified by MTT assay. WJ-MSCs were seeded in a 48-well plate at a density of 1 × 10^4^ cells/well for 6 days. The error bars represent the SD about the mean. Asterisk (*) indicates statistical significance (*p* < 0.05) compared with the m-GO film on the same day.

We then investigated the potential of our modified m-GO film in biomedical applications. WJ-MSCs were cultured on the m-GO film and peptide-conjugated m-GO films (PepS1/m-GO, PepS2/m-GO, PepS3/m-GO, and PepS4/m-GO film) for up to 6 days. To verify the effect of the peptide on the cell morphology, the cells cultured on samples were observed under bright field microscopy (Figure S3). WJ-MSCs grew similarly on the parent m-GO film and the peptide-conjugated m-GO films over the 6 days of proliferation. WJ-MSCs cultured on all samples appeared to be homogenously dispersed on the surface and reached 80% confluency on day 6. The cell growth on all samples maintained a spindle-shaped and fibroblast-like morphology, which is a typical morphological characteristic of WJ-MSCs [71]. In addition, an MTT assay was carried out to confirm the cell viability data after the first, third, and sixth day of the incubation period on the prepared samples. The results of the MTT assay are shown in Figure 3. For all the prepared samples, the MTT results revealed no difference in cell viability after being cultured for the first and third day. However, on the sixth day of culture, the cell viability of the WJ-MSCs on the peptide-conjugated m-GO films was greater than that on the parent m-GO film. This could be attributed to the presence of the peptide, which could enhance the m-GO film biomimetic functions, resulting in better cell binding points to fulfill the role of extracellular matrix support. As previously reported by Katayama and coworkers, a synthetic peptide derived from an enamel matrix derivative was able to promote the growth of human mesenchymal stem cells (hMSCs) throughout the 7-day culture period [49]. The above results collectively confirm that peptides conjugated on the m-GO film did not hamper the normal growth of the WJ-MSCs, but rather provided a high biocompatibility and suitable environment for the proliferation of the WJ-MSCs. 

### 3.3. Osteogenic Differentiation of WJ-MSCs on the Multilayer GO and Peptide/m-GO Films

**Figure 4:**
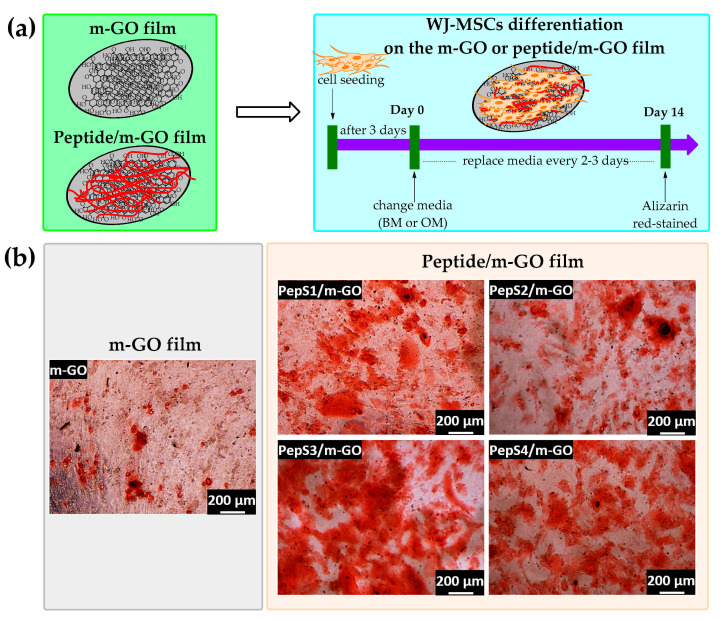
Peptide-conjugated m-GO film promotes osteogenic differentiation of WJ-MSCs. (**a**) Schematic demonstration of the procedure to differentiate WJ-MSCs into osteogenic lineages on the m-GO and peptide/m-GO film. (**b**) Alizarin Red staining of WJ-MSCs on the m-GO film before and after conjugate with peptide sequences (PepS1, PepS2, PepS3, and PepS4) after 14 days of osteogenic medium (OM) incubation. All scale bars represent 200 µm.

Osteogenesis is one of the major differentiation lineages of MSCs that can be directed by engineering the biophysical properties of biomaterials [13,72,73]. Thus, the osteogenic differentiation of WJ-MSCs on m-GO and peptide-conjugated m-GO films was evaluated in the basal medium (BM) and osteogenic medium (OM) (Figure 4a). By day 14, both cells in the BM and OM were stained with Alizarin Red, which serves a visual indicator to detect the formation of osteoblast mineralization (calcium deposits) [19,55]. The experimental data (Figure S4) indicated that the WJ-MSCs cultured on both the m-GO and peptide-conjugated m-GO films maintained a spindle shape for 14 days when cultured in the BM and showed negligible Alizarin Red staining. This suggests that all of the substrates were not able to cause osteogenesis in the BM condition. In other words, the cells maintained undifferentiated behavior under this experimental condition. However, the cells cultured with the OM condition were clearly stained with Alizarin Red on day 14 (Figure 4b). Based on the qualitative data, the stained areas of all of the peptide-conjugated m-GO films were more intense than the parent m-GO film. The presence of the peptide sequence derived from the extracellular matrix (ECM) can provide adhesive interactions, which are critical for stem cell maintenance within the niche and encourage growth [33,72,74]. Qi and coworkers assembled a polypeptide (poly-L-lysine, PLL) coupled with a GO film using a layer-by-layer technique (GO/PLL film). The researchers observed a high preconcentration capacity of osteogenic chemicals (dexamethasone, β-glycerol phosphate, and ascorbic acids) through the *π–π* stacking and electrostatic and hydrogen bonds of the GO/PLL film, resulting in a great enhancement of the osteogenic differentiation of rat-bone-marrow-derived MSCs as compared with GO (GO coverslip) alone [32]. Our peptide-conjugated m-GO films accompanied by OM were able to provide a biocompatible platform to facilitate cell–substrate interactions, resulting in the enhanced osteogenic differentiation of WJ-MSCs. To the best of our knowledge, the current study provides pioneer evidence for the use of peptide-conjugated m-GO films as a potential biomaterial for growing and inducing osteogenic differentiations of WJ-MSCs. 

## 4. Conclusions

In conclusion, a new type of peptide/m-GO films was fabricated through mild and inexpensive self-assembly. The fabricated peptide/m-GO films (PepS1/m-GO, PepS2/m-GO, PepS3/m-GO, and PepS4/m-GO film) possessed similar oxygenated groups to the parent m-GO film, with an extra amide peak derived from peptide. The peptide conjugate on the m-GO films promoted the WJ-MSC proliferation and enhanced the osteogenic differentiation based on qualitative analysis. Overall, the peptide/m-GO films could have a promising role in the significant enhancement of controlling the osteogenic lineage of WJ-MSCs for future application in bone regeneration, as well as tissue engineering, due to their easy fabrication process and biocompatibility. However, further studies with quantitative analysis, such as alkaline phosphatase (ALP) activity quantification, calcium content quantification, and gene expression analysis of osteogenic markers by RT-qPCR, are required to further confirm the effects of peptide/m-GO films on WJ-MSCs. In vivo studies are also required to confirm the hypothesis for clinical application.

## Figures and Tables

**Table 1:** Characteristics of designed peptide sequences.

Code	Short Bioactive Peptide Sequence	Number of Positively Charged Amino Acids	Number of Aromatic Rings Amino Acids	Reference
PepS1	YIGSRWYQNMIRIKVAV	3 (Arg, Arg, Lys)	3 (Trp, Tyr, Tyr)	[45,49]
PepS2	QHREDGSYIGSRIKVAV	4 (His, Arg, Arg, Lys)	2 (His, Tyr)	[45,50]
PepS3	WQPPRARIYIGSRIKVAV	4 (Arg, Arg, Lys, Arg)	2 (Trp, Tyr)	[45,48]
PepS4	DGEARGDSPKRSR	4 (Lys, Arg, Arg, Arg)	0	[45,46,47,51]

**Table 2:** Surface composition of the fabricated GO and GO/peptide films.

Element			Weight (%)		
	m-GO Film	PepS1/m-GO Film	PepS2/m-GO Film	PepS3/m-GO Film	PepS4/m-GO Film
C	34.48	42.96	45.64	21.00	29.59
O	65.52	55.04	52.23	74.70	66.80
N	0.00	2.00	2.13	4.30	3.61
Total	100	100	100	100	100

## Data Availability

All the data will be available to the readers.

## Supplementary Materials

The following are available online at https://www.mdpi.com/article/10.3390/polym13193290/s1, Figure S1: The 3D AFM topographic image of m-GO film (a) before and (b–e) after conjugation with peptide at 2.5 µm × 2.5 µm, Figure S2: The raw FTIR spectra of glass, m-GO film and functionalized m-GO film with PepS1, PepS2, PepS3 and PepS4. Peaks below wavenumber 1500 cm^−1^ belong to background signal from glass substrate, Figure S3: Morphology of cultured WJ-MSCs onto m-GO film before and after conjugate with peptide sequence (PepS1, PepS2, PepS3 and PepS4) on days 1, 3 and 6. The red arrows indicate WJ-MSCs adhered on substrates. Scale bar represents 200 µm, Figure S4: Alizarin red-staining of WJ-MSCs on m-GO film before and after conjugate with peptide sequence (PepS1, PepS2, PepS3 and PepS4) after 14 days of basal medium (BM) incubation. All scale bars represent 200 µm.
