# Supplementary material for: Peptide Conjugate on Multilayer Graphene Oxide Film for the Osteogenic Differentiation of Human Wharton’s Jelly-Derived Mesenchymal Stem Cells
Source: Polymers (Basel). 2021 Sep 26;13(19):3290. doi: 10.3390/polym13193290 (PMC8512023; doi:10.3390/polym13193290)
Supplement: Supplementary file 1 [file polymers-13-03290-s001.zip › polymers-1356368-supplementary.pdf]

Peptide conjugate on multilayer graphene oxide film for the osteogenic differentiation of human Wharton's jelly-derived mesenchymal stem cells

Perng Yang Puah [1,2,3], Pak Yan Moh [2,*], Coswald Stephen Sipaut [4], Ping Chin Lee [1,5] and Siew Eng How [2,*]

[1] Programme of Biotechnology, Faculty of Science and Natural Resources, Universiti Malaysia Sabah, Jalan UMS, 88400 Kota Kinabalu, Malaysia; pypuah@hotmail.com (P.Y.P.); leepc@ums.edu.my (P.C.L.)
[2] Programme of Industrial Chemistry, Faculty of Science and Natural Resources, Universiti Malaysia Sabah, Jalan UMS, 88400 Kota Kinabalu, Malaysia; pymoh@ums.edu.my
[3] Faculty of Medicine and Health Sciences, Universiti Malaysia Sabah, Jalan UMS, 88400 Kota Kinabalu, Sabah, Malaysia.
[4] Programme of Chemical Engineering, Faculty of Engineering, Universiti Malaysia Sabah, Jalan UMS, 88400 Kota Kinabalu, Sabah, Malaysia; css@ums.edu.my
[5] Biotechnology Research Institute, Universiti Malaysia Sabah, Jalan UMS, 88400 Kota Kinabalu, Sabah, Malaysia.
* Correspondence: sehow@ums.edu.my ; pymoh@ums.edu.my

Supplementary Material

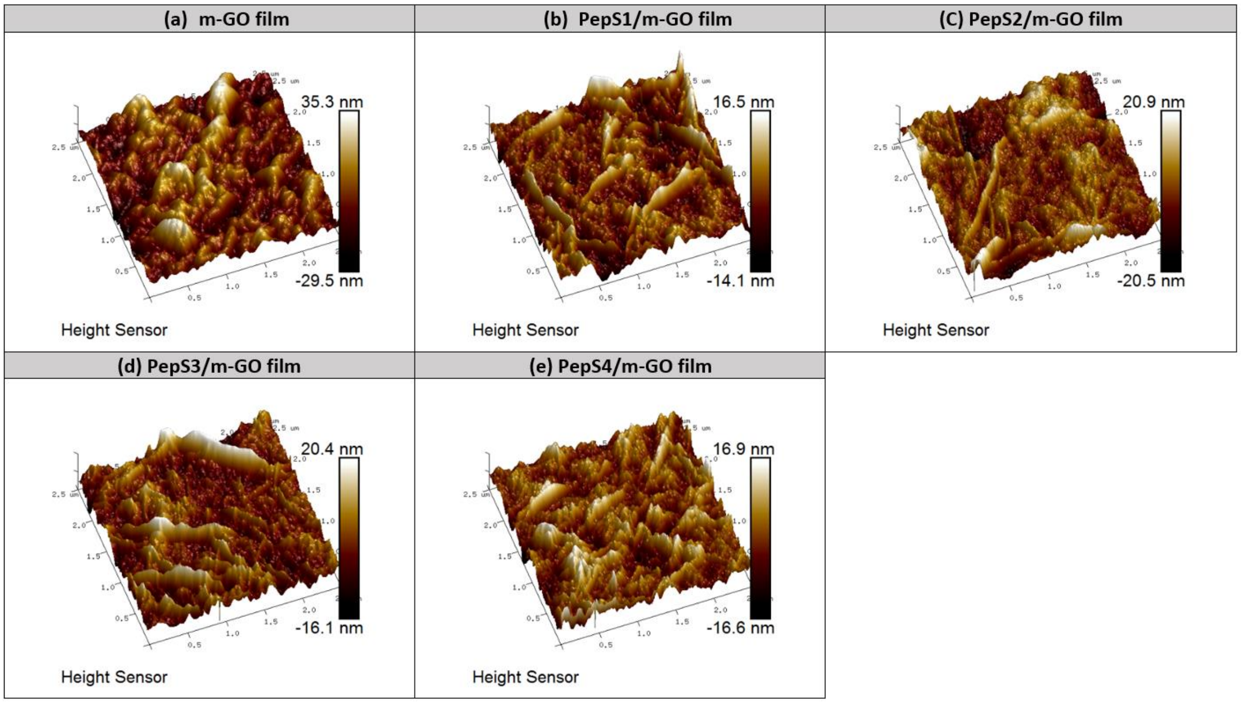

Figure S1: The 3D AFM topographic image of m-GO film (a) before and (b-e) after conjugation with peptide at 2.5 μm x 2.5 μm.

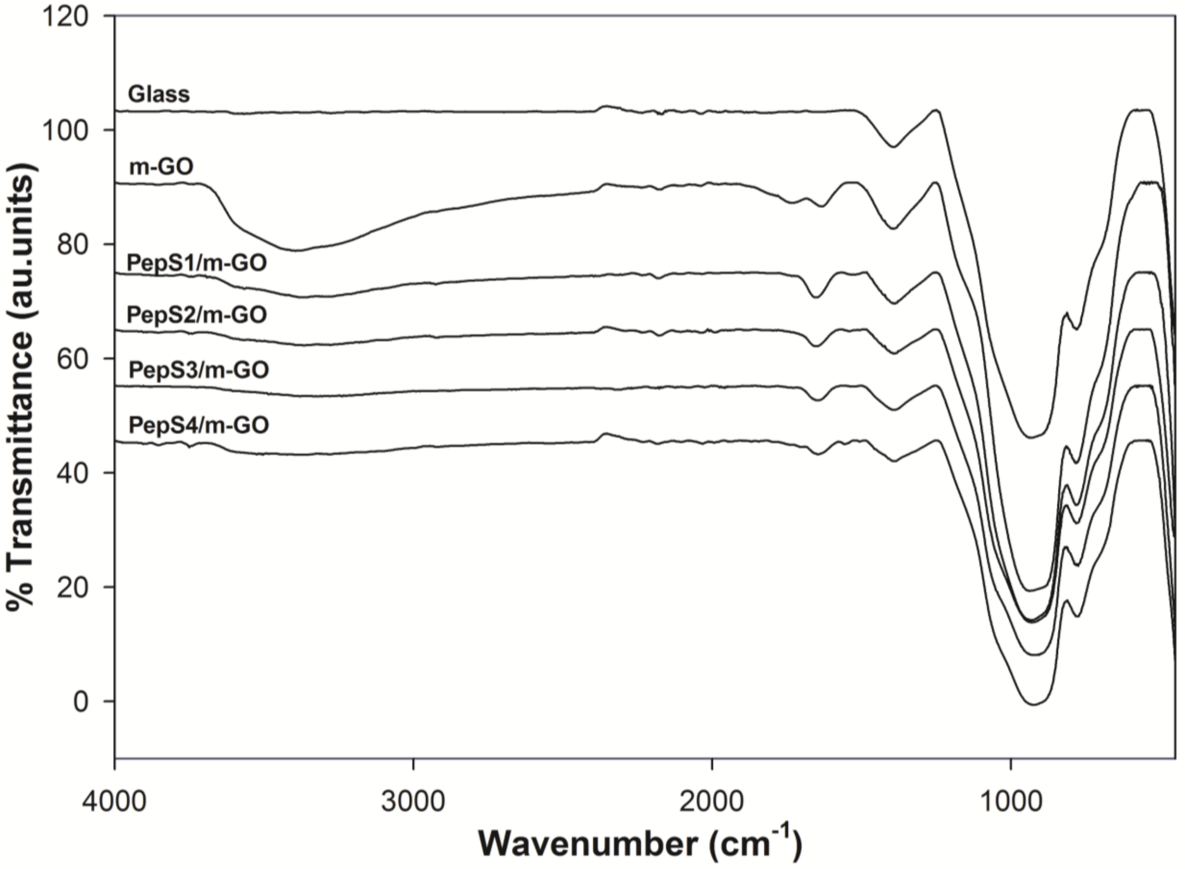

Figure S2: The raw FTIR spectra of glass, m-GO film and functionalized m-GO film with PepS1, PepS2, PepS3 and PepS4. Peaks below wavenumber 1500 cm^{-1} belong to background signal from glass substrate.

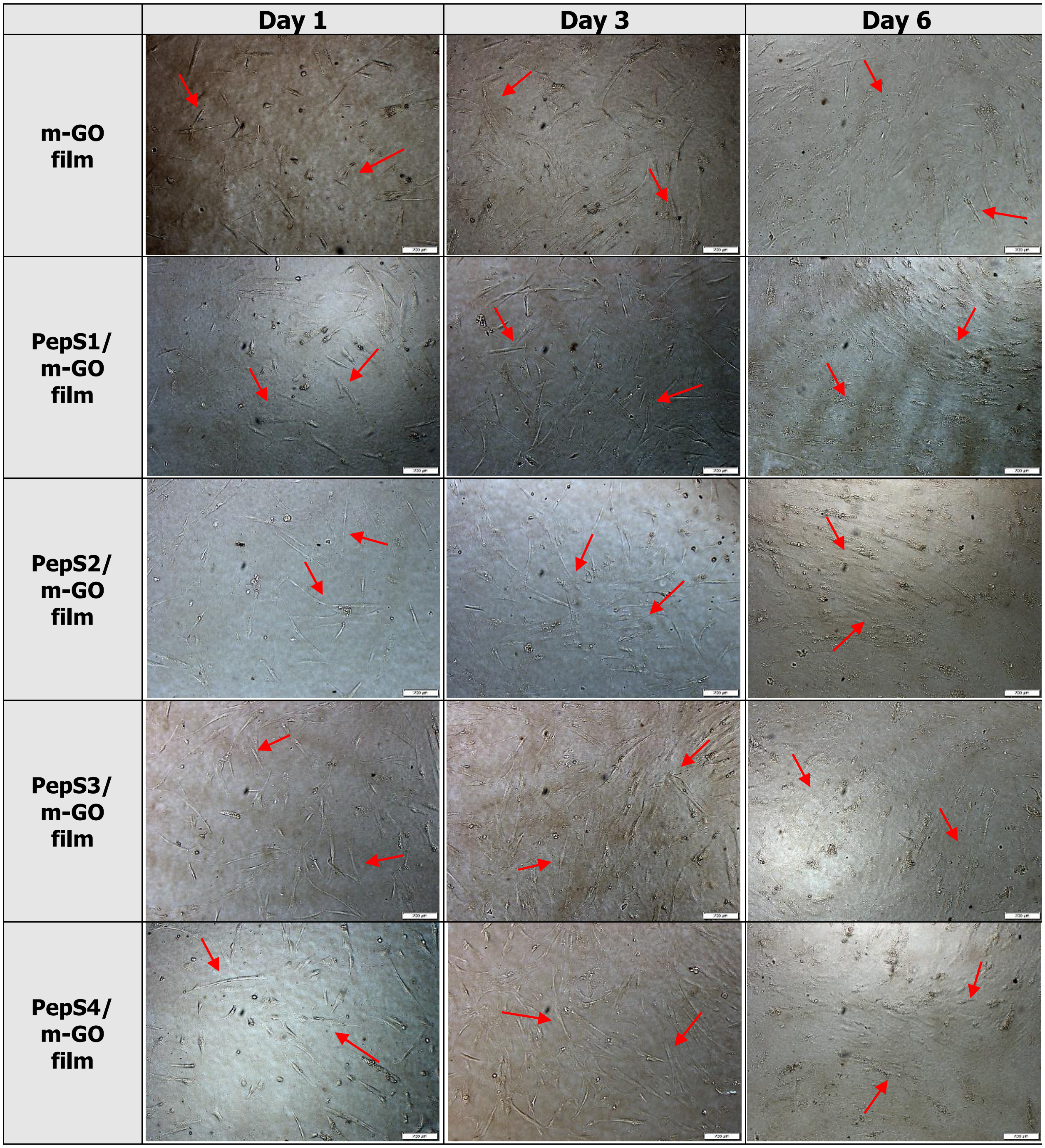

Figure S3: Morphology of cultured WJ-MSCs onto m-GO film before and after conjugate with peptide sequence (PepS1, PepS2, PepS3 and PepS4) on days 1, 3 and 6. The red arrows indicate WJ-MSCs adhered on substrates. Scale bar represents 200 μm.

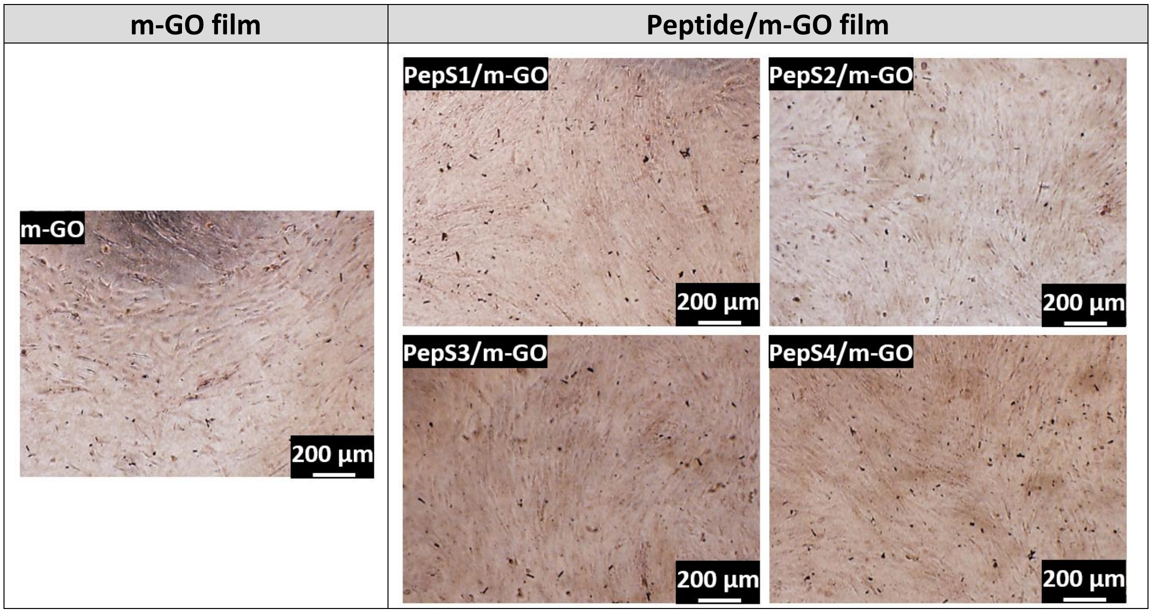

Figure S4: Alizarin red-staining of WJ-MSCs on m-GO film before and after conjugate with peptide sequence (PepS1, PepS2, PepS3 and PepS4) after 14 days of basal medium (BM) incubation. All scale bars represent 200 μm.
